# Transforming Skin Quality Evaluation With AI: From Subjective Grading to Data‐Driven Precision

**DOI:** 10.1111/jocd.70371

**Published:** 2025-08-23

**Authors:** Rainer Pooth, Sonja Sattler, Frederic Westerberg, Tatjana Pavicic, Martina Kerscher

**Affiliations:** ^1^ Clinical Research and Development ICA Aesthetic Navigation GmbH Frankfurt Germany; ^2^ Rosenparkklinik GmbH Darmstadt Germany; ^3^ Private Practice, for Dermatology & Aesthetics Munich Germany; ^4^ Division of Cosmetic Sciences and Aesthetic Dermatology University of Hamburg Hamburg Germany

**Keywords:** aesthetic assessment, artificial intelligence, objective analysis, skin quality, subjective evaluation

## Abstract

**Background:**

Skin quality has a significant influence on aesthetic perception, yet its clinical evaluation remains subjective and inconsistent. Traditional assessments, such as visual grading and manual scoring, lack reproducibility and fail to capture subtle changes over time.

**Aims:**

To explore how artificial intelligence (AI) can transform skin quality evaluation by introducing objective, data‐driven metrics that enhance precision, reproducibility, and personalization in aesthetic medicine.

**Methods:**

We conducted a narrative review of the literature on AI‐based skin analysis tools and their role in quantifying key skin quality dimensions, including pigmentation, texture, elasticity, radiance, and erythema. Emphasis was placed on the use of standardized imaging, emergent perceptual categories (EPCs), and composite scoring systems designed to capture multidimensional aspects of skin quality.

**Results:**

AI tools enable the objective quantification of skin quality through high‐dimensional image analysis, thereby reducing interobserver variability and supporting consistent evaluation across time points and populations. These systems facilitate longitudinal monitoring, tailored interventions, and patient‐clinician communication. By integrating individual demographics and environmental variables, AI fosters equitable and personalized care. Regulatory and ethical considerations, such as data privacy and algorithmic bias, must be addressed to ensure the responsible implementation of these tools.

**Conclusions:**

AI represents a paradigm shift in aesthetic dermatology, offering standardized and reproducible metrics for assessing and monitoring skin quality. When aligned with validated frameworks, such as the EPCs, AI supports improved treatment outcomes, patient satisfaction, and industry‐wide standardization. Future progress depends on interdisciplinary collaboration, robust regulation, and inclusive data practices.

## 1 Introduction

The concept of skin quality has gained prominence in both aesthetic medicine and general healthcare. Its pivotal role in shaping the overall aesthetic perception of the face has been well established over the years [1, 2]. As a key determinant of perceived health, age, and attractiveness, skin quality contributes to psychosocial well‐being and treatment satisfaction [2, 3, 4, 5]. Minor variations in skin tone or texture can substantially impact the facial aesthetic perception across all ethnicities and genders [3, 4, 6, 7]. Age‐related changes, such as decreased elasticity due to collagen and elastin loss, diminished hydration levels, and uneven skin tone and pigmentation, further underscore the need for comprehensive skin quality evaluation [8, 9].

Effective treatment outcomes depend on viewing the skin holistically, addressing the epidermis, dermis, and subcutaneous layers through a multimodal approach. This includes resurfacing, hydration, collagen stimulation, and volume restoration, all of which aim to improve skin quality by enhancing texture, tone, and elasticity [10, 11, 12, 13, 14]. Tracking the effects of these treatments on skin quality is essential, and standardized, objective metrics should be used to assess progress thereof to provide effective treatments.

According to a global consensus, skin quality is best described by four key emergent perceptual categories (EPCs): *skin tone evenness*, *skin surface evenness*, *skin firmness*, and *skin glow*. Each EPC includes multiple constituents—for example, skin tone evenness is determined by pigmentation, erythema, and discoloration. This structured framework provides a shared clinical language for understanding and evaluating changes in skin appearance over time [2]. However, despite the growing clinical relevance of Skin Quality and EPCs, the tools available for evaluating them in practice remain limited. Unlike other aspects of facial aesthetics, such as volume, symmetry, or contour, that can often be measured using calibrated imaging or standardized devices, skin quality lacks universally accepted, objective, and reproducible metrics. Most assessments still rely on visual inspection or manually applied grading scales, which are inherently subjective and prone to interobserver variability [15]. This subjectivity challenges consistency across clinicians and settings, particularly when monitoring subtle shifts over time or comparing results across studies. While these separate assessments of the EPCs provide valuable insights, a unified approach is needed. Combining these EPCs into a standardized score, such as a Skin Quality Index (SQI), has been proposed to provide a holistic, objective, and reproducible assessment. Artificial intelligence (AI) presents a transformative solution, addressing the limitations of traditional methods by providing objective, data‐driven insights.

In this context, AI enables the extraction and analysis of high‐dimensional visual data to quantify skin features with greater precision, consistency, and reproducibility [16, 17].

This paper explores the integration of AI in the evaluation and treatment of skin quality, focusing on the development of novel, AI‐driven metrics, personalization strategies, and the regulatory considerations required for their application. Through the use of real‐time, comprehensive, and reproducible data, AI‐based tools have the potential to transform aesthetic medicine. Examples of emerging tools, including those integrating skin quality scoring models, illustrate how the EPCs may be operationalized. These references to scoring models, such as the SQI, are illustrative examples of how AI might support standardized assessment.

## 2 AI & Skin Quality: A Paradigm Shift

Traditional methods for assessing skin quality, such as manual grading of pigmentation, hydration, elasticity, and surface texture, have long been the standard for aesthetic practitioners [18, 19, 20, 21]. While these approaches are easily and widely accessible, they are also inherently subjective, resulting in variable results, particularly in multi‐center or international settings where differences in training and cultural standards can further skew assessments [22, 23]. Device‐based methods, including cutometry or transepidermal water loss measurements, provide valuable and precise data that are often considered quantitative gold standards in research and clinical diagnostics. However, such measurements are often time‐consuming, equipment‐dependent, and impractical for routine daily aesthetic practice use [24, 25, 26, 27].

It is important to clarify that physical measurement techniques and AI‐based image analysis address different aspects of skin assessment. Physical methods provide direct quantification of biomechanical or physiological properties that cannot be captured through imaging alone. Rather, AI enables the extraction of visual features related to skin tone evenness (e.g., pigmentation patterns, erythema), surface evenness (e.g., texture and roughness), and firmness (e.g., contour definition). This enables consistent evaluations and minimizes human bias already demonstrated in various medical specialties [28, 29, 30], offering a complementary, scalable solution in routine aesthetic practice.

**FIGURE 1:**
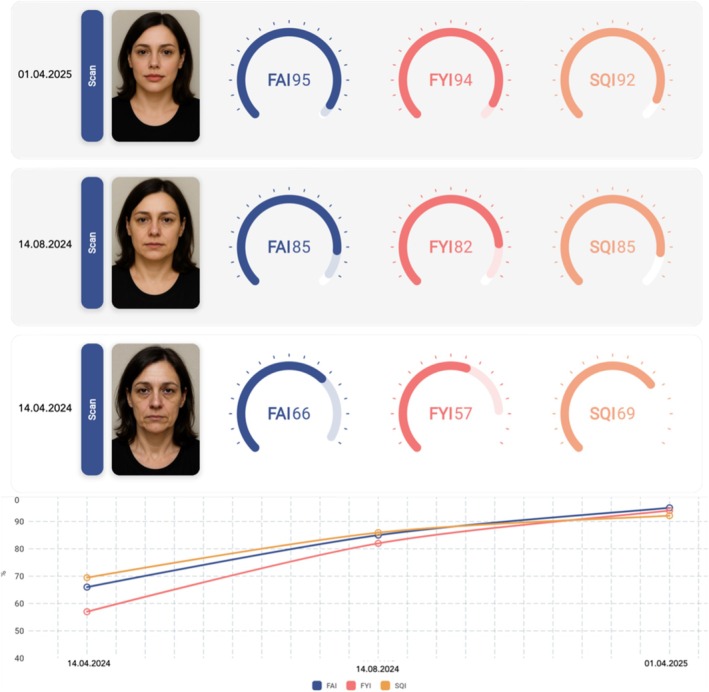
AI‐driven longitudinal analysis of aesthetic and skin quality metrics using proprietary scoring tools. Serial assessments of the Facial Aesthetic Index (FAI), Facial Youthfulness Index (FYI), and Skin Quality Index (SQI), as generated by a proprietary platform (Caarisma, ICA Aesthetic Navigation GmbH), are shown in a 45‐year‐old female patient at baseline (bottom), after the first consultation (middle), and after the second consultation (top). A corresponding longitudinal chart illustrates progressive improvements across all indices over time. This example is included to illustrate how AI‐driven image analysis may support structured tracking of aesthetic and skin quality outcomes following multimodal therapy.

To support standardized and longitudinal assessment of these parameters, proprietary tools such as the SQI have been developed. The SQI consolidates outputs related to EPCs into a unified score that can be tracked over time to evaluate treatment response. This illustrates how AI‐enabled scoring models may support more objective clinical decision‐making and enhance communication between practitioners and patients (Figure 1). Further examples of emerging AI tools include the Facial Aesthetic Index and Facial Youthfulness Index which—similar to the SQI—use image‐derived metrics to assess various parameters culminating in one holistic easy‐to‐interpret score [31, 32]. Commercial platforms such as Haut.AI and ModiFace's SkinConsult AI analyze standardized facial images to evaluate features like tone evenness, radiance, and fine lines. Similarly, Perfect Corp's AI Skin Scanner and other mobile‐based tools use deep learning to detect wrinkles, redness, and dullness across different skin types. While some of these tools are already commercially available and widely used in aesthetic or consumer applications, others remain investigational and are primarily applied in academic research settings. The SQI is already used for standardized image‐based treatment planning in clinical practice.

Beyond delivering precise evaluations, AI allows for the dynamic, longitudinal analysis of skin quality parameters, factoring in individual aging patterns, treatment responses, or environmental impacts. Such monitoring enables practitioners to detect early‐stage deficiencies and deviations from baseline skin quality. With standardized imaging protocols, such systems can compare data across visits, reducing subjectivity and supporting real‐world outcomes measurement. Moreover, AI can ensure equitable access to consistent skin quality assessment and treatment recommendations regardless of geography, practitioner, or patient demographics [33, 34, 35, 36, 37].

AI holds potential to offer predictive insights into aesthetic outcomes for surgical and non‐surgical treatments, including injectables, biostimulators, and energy‐based devices (EBDs), enhancing treatment planning and boosting patient satisfaction [38, 39] (Figure 1).

## 3 Improving Skin Quality Metrics With AI

AI is reshaping aesthetic medicine by introducing standardized metrics to evaluate skin quality objectively. Rather than relying on subjective impressions, AI uses advanced algorithms to assess and quantify parameters such as pigmentation, radiance, and dullness, wrinkle depth, surface texture, elasticity, and erythema, which can be mapped to the four key components within the EPC framework: skin tone evenness, surface evenness, firmness, and glow. These parameters are derived from high‐resolution facial images and include features such as local texture variation, pigment density, shadow mapping, wrinkle fold depth, and distribution of erythema. The extracted features are algorithmically processed to enable consistent, objective classification across the EPCs.

Eliminating the subjectivity of manual assessments provides practitioners with a standardized, reliable tool for precise skin quality evaluation, ensuring consistent and comparable results across patients and practices [15, 31, 32]. While some AI tools generate multiple outputs corresponding to distinct skin quality parameters, such as pigmentation irregularity, wrinkle density, or erythema distribution, some systems compute a composite index (e.g., the SQI) that aggregates key metrics into a single standardized score, thereby supporting a holistic assessment over time.

For AI to be clinically effective, the implementation of standardized imaging protocols before and after treatment sessions is crucial. Some platforms analyze over 1000 variables to support personalized treatments, track outcomes over time, and enhance patient‐practitioner communication [31, 32].

One of AI's most transformative contributions is its ability to apply standardized algorithms to different demographic groups and clinical settings. By accounting for variations in skin features influenced by ethnicity, age, and environmental factors, AI enables precise and equitable evaluations of skin quality [40, 41, 42]. This consistency minimizes human bias, bridges gaps in clinical experience with different skin types, and enhances reproducibility across practices. As a result, AI‐driven skin quality assessments can foster confidence in treatment protocols, support the establishment of industry‐wide standards, and provide practitioners with reliable data to develop personalized interventions. In addition to point‐in‐time assessment, AI's capacity to track skin changes over time enables practitioners to evaluate the treatment efficacy with greater objectivity. By comparing high‐dimensional imaging data over time, clinicians can detect subtle deviations from baseline conditions, assess the progression or reversal of age‐related changes, and dynamically adjust treatment plans based on patient response. This real‐time adaptability may help prevent overtreatment while optimizing outcomes, particularly when managing multimodal protocols involving injectables, biostimulators, or EBD's.

Looking ahead, AI's integration with regenerative modalities, such as exosome‐based treatments or neocollagenesis, may elevate its role in personalized aesthetic medicine. As these tools evolve, their alignment with the EPC framework ensures that innovation remains grounded in clinically relevant dimensions in skin quality [2].

## 4 Personalization Through AI

Skin is inherently unique, shaped by a complex interplay of genetics, environmental exposure, and the recently uncovered role of epigenetic modifications. As one example of skin individuality, epigenetic changes significantly influence skin aging, affecting key attributes such as firmness, elasticity, and pigmentation [43, 44, 45]. Given this variability, effective skin care demands reproducible and objective measures capable of adapting to each individual's distinct characteristics.

AI has revolutionized personalization in aesthetics by enabling highly tailored treatment plans based on detailed analysis of individual skin features, genetic predispositions, and environmental influences. The availability of high‐dimensional datasets allows AI to analyze a wide array of skin quality parameters, demographic information, and lifestyle factors. This wealth of data empowers AI to move beyond generic treatment protocols, offering bespoke, patient‐specific recommendations that are optimized for each individual's unique needs. By integrating vast datasets that include skin texture, pigmentation, and hydration indicators alongside lifestyle factors such as diet, stress, and environmental exposure, AI can generate precise recommendations that move beyond generic “one‐size‐fits‐all” approaches. These insights empower clinicians to design bespoke treatment protocols, incorporating targeted topicals, injectables, biostimulators, and EBDs to address each patient's specific needs. For example, AI can identify deficiencies in visible skin quality attributes and volume loss, informing the use of interventions such as hyaluronic acid injections, which improve hydration, elasticity, and firmness by enhancing the extracellular matrix [46, 47].

One of AI's most powerful advantages is its ability to support dynamic treatment monitoring by assessing changes in visible skin parameters over time, thereby positioning itself as a digital companion in the aesthetic journey of the patient [47]. For example, AI can detect subtle changes in surface texture or pigmentation patterns that may reflect evolving skin quality [48]. However, parameters such as hydration or elasticity are traditionally assessed through physical measurement devices, and current AI imaging alone may not fully capture these attributes. Recognizing these limitations, AI's role is positioned as a complementary tool that enhances maintenance strategies by tracking visual indicators, supporting clinicians in fine‐tuning treatment protocols based on patient responses.

By addressing deficiencies before they become clinically significant, AI can also serve a preventative approach that optimizes outcomes while reducing overtreatment risks. AI also enhances maintenance protocols by enabling clinicians to fine‐tune treatment plans based on a patient's response over time. This precision maximizes the efficacy of interventions, ensuring safer and more effective care.

AI‐powered platforms empower clinicians and provide consumers with user‐friendly interfaces offering personalized insights into their skin quality. By anchoring these personalized insights within the established EPCs, AI ensures that patient education and engagement are directly connected to measurable improvements in tone evenness, surface evenness, firmness, and glow. Such tools can enable patients to make more informed choices about skincare products and aesthetic treatments, promoting proactive engagement and better adherence to recommended regimens [47, 48, 49]. Visualizing patient progress through detailed, objective metrics further sets a common ground for communication between providers and patients, fostering transparency and trust. By continuously refining treatment strategies, AI stands to elevate the standard of care in aesthetic medicine. Its ability to combine precision, adaptability, and personalization positions AI as an indispensable tool in modern skin quality evaluation and management.

## 5 Regulatory Considerations

The rapid integration of AI into aesthetics introduces significant regulatory challenges, primarily in ensuring safety, efficacy, and data integrity [50]. While some AI algorithms—particularly those using reinforcement learning—are dynamic systems that adapt during deployment and require careful regulatory oversight, some models used in aesthetic skin quality analysis are static and do not retrain once implemented. For these static models, performance remains stable unless manually updated, though regular validation across diverse populations remains important to avoid systemic bias [51, 52, 53].

Data privacy remains a critical concern, given the sensitive nature of facial and skin data processed by AI systems. While AI models depend on large datasets to optimize their accuracy, the collecting, storing, and analyzing of such data carry inherent privacy risks. Regulations must enforce encryption, anonymization, and secure storage protocols to safeguard sensitive patient information [54]. Clear and transparent informed consent processes are also necessary to ensure that patients understand how their data will be used, shared, and retained. Training data for aesthetic AI systems are commonly sourced from clinical image repositories, standardized photographic datasets, and multicenter collaborations that collect high‐resolution facial images under controlled conditions. To ensure data integrity and regulatory compliance, facial image data must be anonymized, encrypted, and accompanied by clear, informed consent outlining its intended use. Furthermore, compliance with regulations such as the General Data Protection Regulation (GDPR) is essential for lawful data processing. Establishing transparent data acquisition protocols is critical not only for technical performance but also for building user trust in AI‐enabled aesthetic tools. Ensuring compliance with global privacy laws, including GDPR, is essential to protect against data breaches or misuse [55, 56].

An equally pressing challenge is delineating the boundaries of accountability between AI developers and practitioners. Missteps in AI‐driven recommendations could lead to suboptimal outcomes or even harm, raising complex questions about liability. Regulatory frameworks must address these ambiguities in all fields of medicine, including aesthetic medicine, while maintaining an environment that supports innovation and adoption [57, 58].

Developing international standards for AI in dermatology and aesthetic medicine is crucial to overcoming these challenges. Regulatory bodies such as the US Food and Drug Administration and European CE marking authorities play essential roles in approving AI‐powered medical devices for clinical use. These agencies require rigorous testing and validation to ensure technical accuracy and clinical relevance, demonstrating that AI systems deliver measurable improvements in patient outcomes. Standardization efforts must also establish universal benchmarks for AI algorithms, promoting cross‐platform compatibility and interoperability [59, 60]. Such benchmarks are critical in global aesthetic practices, where practitioners often rely on multiple tools and technologies. Adopting consensus frameworks, such as the EPCs for skin quality, would further ensure that AI tools, regardless of developer, align with clinically validated parameters.

Furthermore, addressing potential biases in AI algorithms is essential. Training systems on diverse patient datasets prevents skewed recommendations and helps bridge gaps in healthcare access. In addition, bias can also arise during the labeling process. Even when diverse image datasets are used, aesthetic attributes such as glow, evenness, or radiance are inherently subjective and may be interpreted differently by annotators depending on cultural, professional, or personal frameworks. These discrepancies can introduce annotation bias, which may be unintentionally learned and amplified by the model. Several strategies can mitigate these sources of bias. Multi‐rater consensus labeling, standardized annotation protocols, and inclusion of culturally calibrated assessment frameworks can reduce subjective variability during dataset preparation. Regular algorithm audits and performance benchmarking across demographic subgroups are essential to detect and correct unintended biases. Inclusive regulatory frameworks are necessary to ensure that AI technologies serve all demographic groups effectively, promoting fairness and equity in aesthetic medicine [61, 62]. In this context, a critical ethical consideration in AI‐driven aesthetic medicine is the risk of promoting uniform ideals of beauty. Since AI systems are often trained on large datasets, there is a possibility that the recommendations generated may converge toward homogenized aesthetic standards, potentially reducing individuality. This could unintentionally limit the expression of diverse beauty norms, undermining personal identity and cultural preferences. To mitigate this, AI tools must be trained on inclusive datasets that reflect broad variations in beauty standards across cultures, age groups, and demographic backgrounds. Safeguarding against the promotion of a singular beauty ideal requires that AI systems respect and preserve individual diversity, allowing for personalized and culturally sensitive treatments.

By addressing these regulatory, privacy, and ethical concerns, the field can ensure the safe, effective, and equitable integration of AI into aesthetics, fostering trust and innovation while protecting patient interests.

## 6 Conclusion

AI is revolutionizing dermatology and aesthetics by introducing objective, standardized metrics based on key visual skin quality factors such as pigmentation, radiation, dullness, skin surface evenness, elasticity, and erythema. By enhancing accuracy, reproducibility, and adaptability, AI empowers clinicians to deliver personalized treatments and helps consumers better understand and manage their skin quality. In clinical settings, standardized protocols for AI‐driven skin assessments, including imaging before and after each treatment session, are necessary to ensure that outcomes are consistently tracked and improvements can be demonstrated to patients. Integrating factors like climate, seasonal changes, and lifestyle into skin health solutions promises truly personalized care, while longitudinal monitoring enables early detection and management of deficiencies, optimizing outcomes aligned with improvements in skin tone evenness, surface evenness, firmness, and glow. The future of skin quality evaluation lies not only in assessing individual parameters but also in integrating these measurements into a unified, objective system like the SQI. By combining multiple metrics into one comprehensive score, AI‐driven systems can provide a complete and accurate picture of skin quality, ensuring better treatment outcomes, improved communication with patients, and more effective personalized care.

Collaboration among AI developers, practitioners, and regulatory bodies is essential to unlock these possibilities. Establishing global standards and fostering diversity and inclusion in AI development will ensure equitable, unbiased applications. Continued research and innovation will further refine AI tools, elevating the standard of care in aesthetics. The future of AI in aesthetics depends on balancing innovation with ethical and regulatory safeguards, ensuring transformative, patient‐centered advancements in skin quality management.

## Author Contributions

All authors made equally significant contributions to the concept, design, and execution of this manuscript.

## Ethics Statement

No human participants or animals were involved in the manuscript. This manuscript represents original work conducted with a commitment to ethical research practices.

## Conflicts of Interest

The authors declare no conflicts of interest.

## Data Availability

Data sharing is not applicable to this article as no new data were created or analyzed in this study.
